# Review and Comparison of Antimicrobial Resistance Gene Databases

**DOI:** 10.3390/antibiotics11030339

**Published:** 2022-03-04

**Authors:** Márton Papp, Norbert Solymosi

**Affiliations:** Centre for Bioinformatics, University of Veterinary Medicine Budapest, 1078 Budapest, Hungary; papp.marton@univet.hu

**Keywords:** antimicrobial resistance genes, antimicrobial resistance gene database, annotation of antimicrobial resistance genes

## Abstract

As the prevalence of antimicrobial resistance genes is increasing in microbes, we are facing the return of the pre-antibiotic era. Consecutively, the number of studies concerning antibiotic resistance and its spread in the environment is rapidly growing. Next generation sequencing technologies are widespread used in many areas of biological research and antibiotic resistance is no exception. For the rapid annotation of whole genome sequencing and metagenomic results considering antibiotic resistance, several tools and data resources were developed. These databases, however, can differ fundamentally in the number and type of genes and resistance determinants they comprise. Furthermore, the annotation structure and metadata stored in these resources can also contribute to their differences. Several previous reviews were published on the tools and databases of resistance gene annotation; however, to our knowledge, no previous review focused solely and in depth on the differences in the databases. In this review, we compare the most well-known and widely used antibiotic resistance gene databases based on their structure and content. We believe that this knowledge is fundamental for selecting the most appropriate database for a research question and for the development of new tools and resources of resistance gene annotation.

## 1. Introduction

Antimicrobial resistance (AMR) means an emerging threat on humanity. Based on a 2017 report, it is estimated that ~ 700,000 deaths can be attributed to AMR worldwide [1]. As stated by a CDC study, approximately 35,000 people die in the United States yearly due to antibiotic resistance [2]. A recent study, however, draws a more drastic picture. Based on data from 2019, approximately 1.27 million deaths can be directly attributed to AMR worldwide [3]. However, it is expected that the impact of AMR will further increase and claim approximately 10 million lives yearly by 2050 [1]. The emergence of resistant microbes will not only cause untreatable primer infections, but the safe performance of routine medical procedures (such as surgeries or chemotherapy treatment of oncological patients) will become impossible due to the inability of a successful antibiotic prophylaxis [1,2,4]. Even though one usually associates AMR with hospitals and the misuse/overuse of antibiotics by medical professionals, the influence of agriculture and the environment is no less important [1,2,4,5,6]. Therefore, to tackle this global challenge the investigation of the spread of AMR between different environments is required.

The genetic background of antibiotic resistance can be categorized into two main mechanisms. On one hand, AMR can arise through genetic mutations (e.g., modification of the antibiotic target site, overexpression of efflux pumps or the antibiotic target molecule etc.), under the selective pressure of antibiotics, or by the acquisition of specific genes conferring resistance (e.g., genes coding enzymes that degrade the antibiotic compounds or open alternative metabolic pathways for evading the effects of the antibiotic) through horizontal gene transfer (HGT) [7,8]. It is believed that the majority of antimicrobial resistance genes (ARG) transmitted between bacteria is not the novel product of widespread antibiotic usage by humans, but has evolved previously for a variety of functions and has been enriched with the extensive usage of antibiotics since the mid 20th century [9,10,11]. As environmental microbes have a significant role in the spread of resistance genes, the global surveillance of ARGs in various environments is critical for understanding and combating AMR [6,11]. As bacterial AMR is currently the most important form of resistance in microbes, we will refer it to when we mention AMR throughout this review.With next-generation genome sequencing (NGS) technologies become widespread in recent years, they are commonly used in AMR surveillance studies either in clinical settings [12,13], or in the agriculture and food industry [14,15,16,17] and the environment [12,18,19]. In line with the importance of genomic surveillance of AMR, several annotation tools and databases have been developed for the analysis of ARG content of bacterial genomes or NGS metagenomic samples [20,21,22,23,24,25,26,27,28,29,30,31,32,33,34,35]. Table 1 presents some information on the most well-known AMR databases.

ARG databases can be divided into two major types [44], some of them contain species specific information (e.g., the MUBI database containing mutations conferring resistance in *Mycobacterium tuberculosis* [45]), while others focus on ARGs from all sources (e.g., the CARD database [21]). However, ARG databases can differ not only in the covered species, but on the type of AMR mechanism as well. Some database specialize only on acquired resistance genes, while others contain only mutations (e.g., the ResFinder [42] database focuses on acquired resistance genes, while the PointFinder [30] database from the same research group covers only AMR associated mutations). Unsurprisingly, however, there are databases with information on both AMR mechanisms (e.g., the CARD [21] or NDARO [34] databases). 

The number of tools and databases focusing on AMR has rapidly grown in recent years, and many review articles were published trying to summarize the information on these resources. However, they put more emphasis on the different tools designed for ARG annotation rather than the databases supplying the information for these tasks [18,44,46,47,48].

As the performance of each tool heavily relies on the underlying database [36], it is important to understand the advantages and limitations of all databases available for the research community. By understanding it, researchers can select the best database for their purpose. Furthermore, this knowledge can be important for choosing the best resource for developing new annotation tools as well. The many available resources of ARGs are not only a blessing, but are a curse as well, as researchers need up-to-date and thorough understanding on them to select the most appropriate one for the task at hand. This can be rather cumbersome as each database differs in structure and logic, especially in the way they store the annotation and metadata associated with ARG sequences. Our main goal is to help such decision making by presenting the comparison of the resources from several aspects. In this review, we compare the most important ARG resources available today. Firstly, we review the structure of each database and then we directly compare them by their content. We present this comparison from the acquired and mutation based resistance mechanisms separately as databases can significantly differ in these regards. Researchers might prefer one mechanism of AMR more in their study, for example, in a study investigating environmental ARGs with potential mobilization properties, acquired resistance genes might be the primary focus, whereas mutations can be more important in a clinical context [49].

## 2. Comparison of the Structure of Databases

### 2.1. Databases Reviewed in this Article

From the databases summarized in Table 1, ARDB, ARG-ANNOT and ResFams are not covered by this review as they are not actively updated (they haven’t been updated since 2008, 2018 and 2015, respectively). Furthermore, Mustard is also not reviewed here as it was constructed for a study of the gut resistome profiling of humans and wasn’t dedicated as a comprehensive resource of ARGs [31]. FARME and PATRIC database are not covered here as well. FARME is based on several metagenomic studies, which were characterized based on their predicted ARG content and AMR phenotype; however, those genes were not extensively validated and might contain false positives [29,48]. PATRIC is constructed for collecting genome sequence data and associated metadata of pathogen microorganisms [35], and necessarily relies on a specialized annotation system for the curation of the data. The ARG annotation pipeline employed by PATRIC is based on the NDARO and CARD databases as well as data from scientific literature, which was reannotated by experts [41]; however, this is not available on their FTP site. Therefore, the following six databases are covered in detail only in this review: ARGminer, CARD, MEGARes, NDARO, ResFinder and SARG.

### 2.2. ARGminer

ARGminer is an ensemble database assembled from several independent ARG resources. It is based on the CARD [21], ARDB [20], DeepARG [50], MEGARes [27], ResFinder [42], and SARG [26] databases [32]. Only the acquired resistance genes were collected from these resources. After the acquisition of the sequences from these databases, they have clustered them to remove duplicates and annotated them by the best match from each of the above data resources. After the assignment of UniProt and GeneOntology metadata to the sequences, they guessed the best nomenclature of each gene name by a machine learning model. However, as several differences can be found between databases, they also utilize a crowdsourcing model to refine annotations (with a trust-validation filter to prevent misuse).

Furthermore, they have collected mobility and pathogen predictions by fitting the sequences to the ACLAME [51] and PATRIC [40] databases, respectively. 

The database is periodically updated with the method described above and published after the verification of ARGminer evaluators. The date of the latest update of the database, at the time of writing of this review, is April 2019.

### 2.3. CARD

The Comprehensive Antibiotic Resistance Database (CARD) is a hand-curated resource that is developed to cover the entire spectrum of ARGs [21]. Every ARG is included in the database based on three criteria. All ARG sequences must be available in the GenBank repository and increase the Minimal Inhibitory Concentration (MIC) in an experimental validation setting which needs to be published in a peer-reviewed journal. Only a handful of historical β-lactam antibiotics are an exception from the above as they do not have an associated, peer-reviewed publication [37]. The CARD database is built around an ontology-driven framework, where the resistance determinants and their associated metadata is recorded in the Antibiotic Resistance Ontology (ARO) network and even the sequences and the threshold used for their detection is stored in a specialized ontology (Model Ontology, MO) [36]. CARD contains resistance genes and resistance mutations as well, which are organized in a species-specific manner. Furthermore, as CARD uses a strict curation procedure for incorporating genes, to increase sensitivity, they have developed a special database (the CARD Resistomes & Variants module) that contains in silico validated ARGs based on the genes stored in CARD [37]. The database is regularly updated based on reviewing the scientific literature by expert curators, whose work is augmented by a machine learning algorithm (CARD*Shark) that sorts scientific publications based on reference for the process. The current version of CARD was updated in October 2021. It is important to note that CARD is freely accessible for academic researchers only, and commercial parties’ use is only permitted with a written license. 

### 2.4. MEGARes

MEGARes are also an assembly of multiple resources in a way that is designed specifically for annotating metagenomic data [27]. The first version of the database was based on ResFinder [42], ARG-ANNOT [22], CARD [21] and the Lahey Clinic β-lactamase database curated by NCBI. During the update of the database to MEGARes 2.0 [38], further sequences were collected from the newer versions of CARD [36] and ResFinder [42] and the NCBI Bacterial Antimicrobial Resistance Reference Database [39]. Furthermore, MEGARes 2.0 also incorporates biocide- and metal resistance genes derived from the BacMet database [52]. After they have removed the duplicates from the sequences collected from these resources, the genes were reannotated which revealed several overlapping genes between the ARG databases and BacMet. As the purpose of the database is to form a basis of the ARG annotation of metagenomic reads that can be used to read abundance based analysis, the annotations are stored in the form of an acyclic graph which avoids that one read or contig is assigned to multiple nodes [27]. The database contains antibiotic resistance genes and mutations as well; however, the mutations are not ordered to microbial species due to the nature of the annotation graph. The current version of the database at the time of the writing of this review was last updated in October 2019.

### 2.5. NDARO

The National Database of Antibiotic Resistant Organisms (NDARO) is a comprehensive database dedicated to antibiotic resistance in the curation of NCBI [34]. The resistance genes are stored in The Reference Gene Catalog, of which, the predecessor was the Bacterial Antimicrobial Resistance Reference Gene Database, with the RefSeq PRJNA313047 BioProject (https://www.ncbi.nlm.nih.gov/bioproject/PRJNA313047) (accessed on: 15 February 2022) storing the reference sequences [39]. This database was constructed from the ResFinder [42], CARD [36], RAC [53] and INTEGRALL [54] databases with extensive curation of the associated scientific literature. Since the expansion of the database in 2021, AMR mutations, general stress response genes and virulence genes are also curated within NDARO for the clinically important pathogens [34]. NDARO is updated regularly; the latest database version was released in December 2021.

### 2.6. ResFinder/PointFinder

ResFinder [42] and PointFinder [30] are dedicated tools for acquired resistance genes and resistance mutations, respectively. These were separate AMR data resources; however, since ResFinder 4.0, they are developed under the same project [33]. ResFinder was originally developed on the basis of the Lahey Clinic β-lactamase database, ARDB [20], and an extensive literature review. To develop a more comprehensive resource of AMR determinants, the developers of ResFinder constructed a database dedicated to mutations conferring resistance only, named PointFinder. During the concatenation of the two databases under the ResFinder 4.0 project, not only was an extensive expert curation applied to the data, but phenotype prediction tables were also constructed to help researchers connect genotype information with potential phenotypic traits. With regular updates, the latest version of ResFinder and PointFinder was released in September and February 2021, respectively.

### 2.7. SARG

The Structured ARG reference database (SARG) is a hierarchically constructed database [26] based on the CARD [21] and ARDB [20] data resources. They only retained the acquired resistance genes from these databases, and after duplicate removal, they have ordered the genes to a two-level hierarchical architecture. The highest level of this hierarchy is the type of the resistance indicating the antibiotic that the genes confer resistance to, while the lower level is the class of the genes. In 2018, the developers of SARG expanded the database by ARG homologs found by aligning the NCBI nt database to SARG [43]. They are regularly updating the database in a similar manner, with the latest aired in January 2022. However, they have not introduced any new ARGs since the 2019 version. SARG, similarly to CARD, is only accessible freely for academic purposes, and a written permit is necessary for commercial use.

## 3. Comparison of the Database Contents

### 3.1. Number of Sequences and ARGs in the Databases

**Figure 1:**
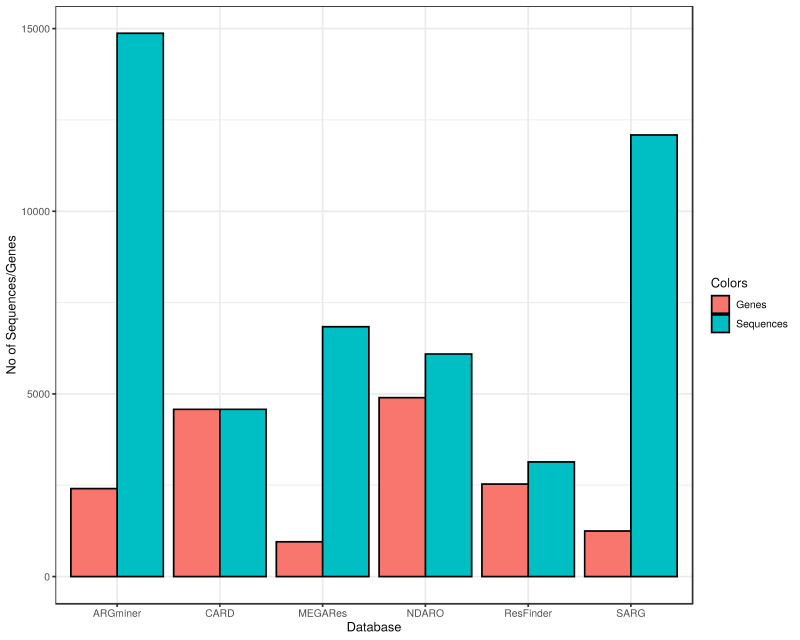
ARG and sequence content of the databases. Only antibiotic and biocide resistance genes were considered for the plot. For each database on the x axis, the number of unique sequences and the corresponding number of unique genes were determined. The y axis represents the number of genes and sequences. Red bars show the gene number while blue bars represent the number of sequences stored in each database.

To compare the ARG content of the different databases, we first matched the number of sequences stored in them and the associated count of unique genes (Figure 1). Figure 1 only shows resistance genes and biocide resistance genes (to maintain comparability between databases), and virulence or metal resistance genes were omitted. The number of unique resistance genes was counted based on the names associated with the particular sequence (i.e., if only the gene family name was given for multiple variants, then only the gene family name was included in the gene count, but if variants had unique names, they were counted separately). In the case of the ARGminer, we have found several different nomenclature forms of the same ARGs, which is not surprising as one of the main goals of the database was to collect and standardize this information with the aid of crowdsourcing. However, as we did not intend to make such standardization through this review, it might be possible that the same gene was counted multiple times in the case of the ARGminer in Figure 1. We tried to reduce the risk of this bias by converting gene names to lowercase when comparing them, as usually the ARG name nomenclature differences concerned only the casing of the letters. Furthermore, we have found 13, 9 and 3 duplicate sequences in the NDARO, ResFinder and MEGARes databases, respectively (the number of sequences in Figure 1 is corrected for the presence of duplicates). The presence of duplicate genes and corresponding sequences in the database might cause overestimation of those genes if the user does not pay enough attention while reviewing the results. In Figure 1, a clear difference can be observed between CARD and the rest of the databases in the relationship between the number of unique sequences and corresponding genes. One might expect that with keeping one reference sequence for each gene, CARD is prone to producing false negatives in homology searches; however, this is overcome in CARD with the use of individual detection threshold for genes stored in the Model Ontology [36].

### 3.2. Gene Count of Antibiotic Classes in Each Database

**Figure 2:**
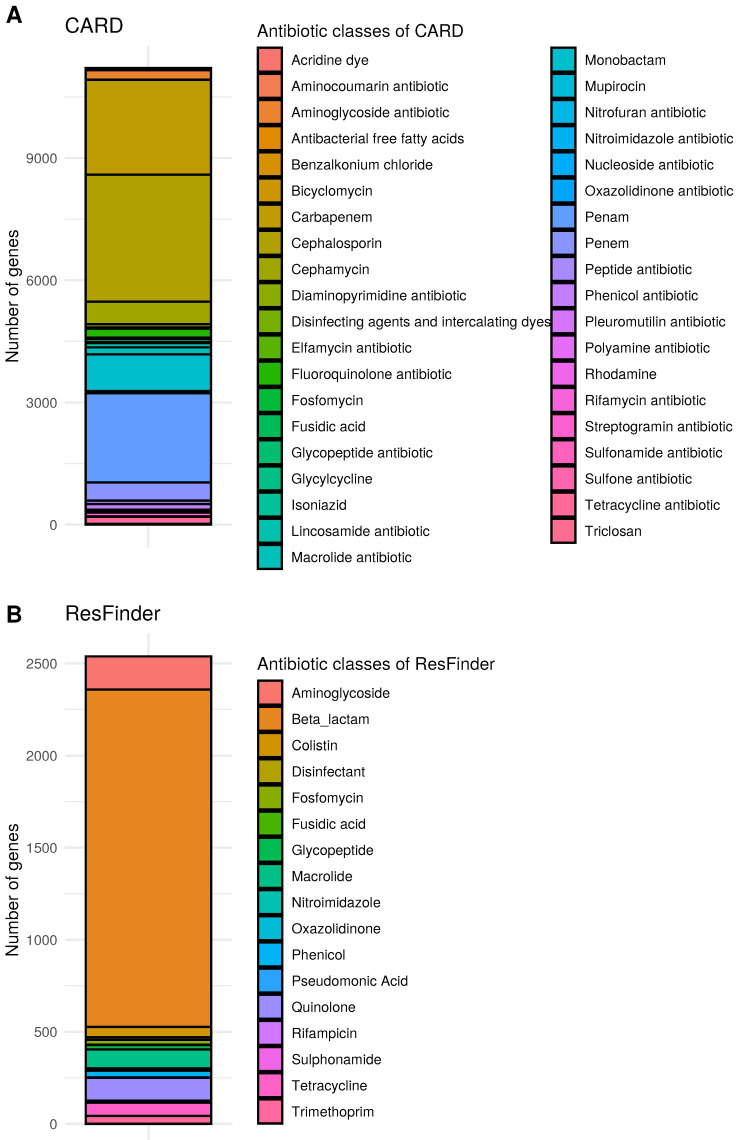
Number of unique genes for each antibiotic class stored in CARD and ResFinder. Bars represent the number of genes in each unique antibiotic or biocide categories, where colors are associated with the specific antibiotics themselves. As one gene can confer resistance to multiple antibiotics, it is possible that the same gene is counted for two or more antibiotics. The plots show the data for CARD (**A**) and ResFinder (**B**), respectively.

Figure 2 shows the differences in the number of antibiotic resistance genes (without those conferring resistance through mutations) associated with the antibiotic classes stored in the respective database for CARD and ResFinder. We have selected these two due to the extensive differences in the depth of the antibiotic classification. For the rest of the databases (MEGARes, NDARO and SARG), the same figures can be found in the Supplementary File S1. In the case of the ARGminer, we could not construct such figure as notable differences were found in some cases between the antibiotic classifications of different records for the same genes. In either of the above figures, the respective classification scheme of each database was used. As one would expect, aminoglycoside and β-lactam antibiotics are the most popular categories in either of the databases. However, there is a significant difference in the classification depth of β-lactams between the CARD and other resources. In CARD, separate β-lactam groups have their respective categories (such as penems, penams, carbapenems, cephalosporins etc.), while others label them only as β-lactams. Furthermore, the presence of several collective categories in the MEGARes database is notable (e.g., multi-drug resistance or drug and biocide resistance, etc.). The reason for the presence of such categories is due to the acyclic form of the MEGARes annotation graph, which does not allow the same gene to link to multiple groups. These figures clearly show that the most comprehensive antibiotic classification of the genes can be expected in the case of the CARD database; however, the differences also emphasize that expert knowledge is important for understanding the results of ARG annotation and one cannot expect to rely entirely on the output of a database.

### 3.3. Microbial Genus with Corresponding AMR Mutations in the Databases

**Figure 3:**
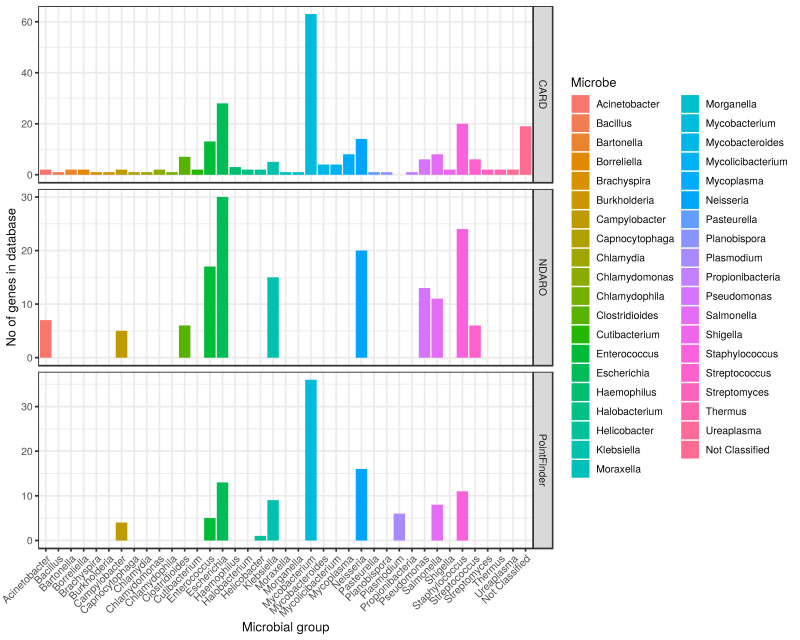
Number of genes conferring resistance through mutations for each microbial genus in CARD, NDARO and MEGARes databases. Genes conferring resistance through mutations was calculated at the genus level for each microbe stored in each database. Only one group could not be summarized at the genus level (propionibacteria). Microbial genus is on the x axis and the number of genes associated with each group in the database is represented by the y axis. Rows show the data separately for each database. Columns are colored by the microbial genus.

Next, we compared the number of genes conferring resistance through mutations for microbial species in each database (Figure 3). Among the databases covered in depth in this review, only CARD, MEGARes, NDARO and PointFinder (element of ResFinder 4.0) comprise such information. Although MEGARes also has information on mutations causing resistance, connecting them to species is not applicable in this case due to the nature of the annotation architecture. In comparing the microbes for which data is stored in each database, we had to find a taxonomical level that can achieve a standardized comparison between all databases. We decided to count genes in the genus level. We had to diverge from this principle only in one case, where the arbitrary group propionibacteria had to be used instead of the corresponding genus. For simplicity, despite this exception, we further refer to the groups of microbes used for the classification in Figure 3 as genus.

It is upfront in Figure 3 that CARD contains mutations for the highest number of genus (37 genuses) between the three databases, and it even has 19 non-species-classified genes as well. In contrast, NDARO store genes for 11 genus while PointFinder stores only for 10. Not every genus considered by the databases belongs to bacteria. CARD stores two genes for *Chlamydomonas* algae and two for the archaea genus *Halobacterium*, while PointFinder has six genes for *Plasmodium* protozoa. Those genus considered by the NDARO and PointFinder databases are primarily human pathogens, especially those among the critically important bacteria for human health, determined by the WHO in 2017 [55,56,57] (ESKAPE pathogens: *Enterococcus faecium*, *Staphylococcus aureus*, *Klebsiella pneumoniae*, *Acinetobacter baumannii*, *Pseudomonas aeruginosa*, and *Enterobacter* species). Furthermore, NDARO has 11 genes enrolled to the *Salmonella* genus, one of the most important foodborne pathogens and often associated with resistance conferring mutations [58]. PointFinder has a significant collection of *Mycobacterium* genes (36 genes), which is even more notable in CARD with 63 genes associated with this genus. Pathogens from the *Mycobacterium* genus, especially *M. tuberculosis* is one of the most important among disease causing bacteria that develops resistance through mutations. Furthermore, as it needs long incubation times for culturing, whole-genome sequencing based approaches are accountable alternatives [59].

CARD database extremely differs from the other data resources reviewed here, due to the high number of microbial genus it collects data for and the number of genes it stores considering AMR mutations. These properties make it especially suitable for AMR mutation screening in a wide variety of study settings; this is even in the case of environmental AMR surveillance as it stores mutations for typical environmental genus such as *Thermus* or *Halobacterium*. A notable number of genes is stored in the database for the *Mycolicibacterium* genus (4 genes), which is in the forefront as a potential bacterium for degrading plastic pollutants [60].

## 4. Conclusions

Previously, several ARG database were constructed to form the bases of ARG annotation of whole-genome sequencing and metagenomic samples. With the advent of NGS, their significance is even more profound, and they became an important augmentation of previous phenotypic screening based studies. We compared the accessible and regularly updated ARG databases in this review, which have new versions released lately. The main focus in this review was on the architecture and content of the different databases, in contrast with previous studies mainly focusing on the tools used for annotation. However, understanding how databases are constructed and the differences between them is crucial for every researcher in the field of AMR, so they can use the most powerful tool for their research question. Based on the differences outlined in this review, it seems that CARD and NDARO are prominent among the databases. NDARO contains the most acquired resistance genes; however, CARD comprises of a similarly high number of genes, making both of them a suitable tool for ARG annotation. In the case of mutations conferring resistance, however, CARD dominates other tools. We advise that in cases where mutations or both type of resistance is considered, CARD should be the number one data resource. Otherwise, choosing NDARO can be a similar or somewhat preferable choice over CARD considering its higher acquired resistance gene content. However, usually one is interested in resistance genes and mutations as well and only in special cases considers acquired resistance only (e.g., when one is interested in environmental resistance determinants possible for transmission to pathogenic bacteria). Furthermore, one should also consider the annotation tool when selecting the most appropriate database. CARD has an advantage in this regard, as its annotation tool (RGI) is accessible through a web interface or can be downloaded as command line software to a computing cluster as well. In contrast, NCBI’s AMRFinderPlus is exclusively accessible as a local tool for linux-based operating systems only, thus requiring specialized bioinformatic skills to operate it. However, not only technical aspects can lead the decision for selecting the most appropriate tool for a study. For example, deep learning approaches are usually considered to be superior in detecting novel resistance gene variants [50], but they rely on the database they were built on (e.g., the latest version of DeepARG was built on the ARGminer database). Although there are annotation tools applicable with any user-defined database [23,28]. The comparison of such tools, however, is beyond the scope of this review. In conclusion, CARD might be the first choice database in most cases, but the best option can differ based on research questions.

Furthermore, the differences in antibiotic classification of the databases emphasize the importance of expert knowledge for interpreting the results. Moreover, as some databases are accessible for non-academic parties only with a written permit, it is important for one to be familiar with the terms of using these resources.

## 5. Future Perspective

We believe that during the evaluation of the performance of different ARG annotation tools, differences in the underlying database should also be considered. Moreover, as major differences can be observed in ARG nomenclature between databases, a standardization procedure would be advantageous for enabling direct comparisons between results generated from different resources. However, such standardization is not only advantageous for the comparability of ARG data resources. One solution for the issue was proposed by ARGminer in the form of crowdsourcing [32] which could standardize the nomenclature within one framework. However, for a unified conclusion, a development of ground rules would be necessary, as was proposed for other issues of ARG nomenclature [61,62].

## Figures and Tables

**Table 1:** Well-known ARG databases. The table contains the most well-known general ARG databases with additional information (the year of the last update of the database (Last Modified), link address, where the database can be accessed (URL) and the publications associated with the database (References).

Database	Last Modified	URL	References
ARDB	Archived, last update 2009	https://ardb.cbcb.umd.edu/ (accessed on: 15 February 2022).	[20]
ARG-ANNOT	Archived, last update: 2018	not available.	[22]
ARGminer *	2019	https://bench.cs.vt.edu/argminer/#/home (accessed on: 15 February 2022).	[32]
CARD *	2021	https://card.mcmaster.ca/ (accessed on: 15 February 2022).	[21,36,37]
FARME	2019	http://staff.washington.edu/jwallace/farme/index.html (accessed on: 15 February 2022).	[29]
MEGAres *	2019	https://megares.meglab.org/ (accessed on: 15 February 2022).	[27,38]
Mustard	2018	http://mgps.eu/Mustard/index.php?id=accueil (accessed on: 15 February 2022).	[31]
NDARO *	2021	https://www.ncbi.nlm.nih.gov/pathogens/refgene/ (accessed on: 15 February 2022).	[34,39]
PATRIC	2017	https://patricbrc.org/ (accessed on: 15 February 2022).	[35,40,41]
ResFams	2015	http://www.dantaslab.org/resfams (accessed on: 15 February 2022).	[24]
ResFinder/PointFinder *	2021	https://cge.cbs.dtu.dk/services/ResFinder/ (accessed on: 15 February 2022).	[30,33,42]
SARG *	2019	https://smile.hku.hk/SARGs# (accessed on: 15 February 2022).	[26,43]

* Considered in this review.

## Data Availability

Not applicable.

## Supplementary Materials

The following supporting information can be downloaded at: https://www.mdpi.com/2079-6382/11/3/339/s1, File S1: Gene counts for antibiotic classes in MEGARes, NDARO and SARG databases.
