# Correction: Papp, M.; Solymosi, N. Review and Comparison of Antimicrobial Resistance Gene Databases. *Antibiotics* 2022, *11*, 339

**DOI:** 10.3390/antibiotics11091169

**Published:** 2022-08-30

**Authors:** Márton Papp, Norbert Solymosi

**Affiliations:** Centre for Bioinformatics, University of Veterinary Medicine Budapest, 1078 Budapest, Hungary

## Text Correction

While preparing our next manuscript, we noticed a few errors in our published paper [1] that we would like to correct.

When determining the unique sequences in the databases, we mistakenly omitted the genes encoding biocide resistance in the NDARO database, which makes the exact number of sequences 6092 instead of 6035. In addition, in the ResFinder database, we found nine duplicate sequences, thus the number of unique sequences is 3137 instead of 3146.

When examining the gene content of each antimicrobial resistance gene database, we counted the unique genes that were given a separate name within the database. This is because it allowed us to compare them despite the structural differences between the databases. We noticed, however, that we counted these genes incorrectly in the NDARO and ResFinder databases. In the NDARO database, we counted individual genes based on Refseq and GenBank identifiers, which do not overlap with the names of genes and variants. In the ResFinder database, the gene names in the header of the fasta files contain the NCBI accession identifiers of the variants as well; here, the error was caused by counting the individual genes together with these identifiers. According to the correction, there are 4898 and 2532 genes in the NDARO and ResFinder databases, respectively.

The number of genes belonging to the antibiotic groups stored in each database has been recalculated accordingly. In addition to this, we noticed that for the CARD database, the categories Rhodamine, Disinfecting agents and intercalating dye and Acridine dye were omitted from the figures.

In addition, for the CARD and NDARO databases, the number of genes for each taxonomic category that confer resistance through mutations was miscalculated for several taxa. We have corrected these calculations.

Accordingly, the following corrections have been made:

1. Section 3.1: the following is inserted after the second sentence to clarify the method of counting genes: “The number of unique resistance genes was counted based on the names associated with the particular sequence (i.e., if only the gene family name was given for multiple variants, then only the gene family name was included in the gene count, but if variants had unique names, they were counted separately).”

2. Section 3.1: The sixth sentence was originally “Furthermore, we have found 13 and 3 duplicate sequences in the NDARO and MEGARes databases, respectively (the number of sequences in Figure 1 is corrected for the presence of duplicates).”. We have modified it to the following ”Furthermore, we have found 13, 9 and 3 duplicate sequences in the NDARO, ResFinder and MEGARes databases, respectively (the number of sequences in Figure 1 is corrected for the presence of duplicates).”

3. Section 3.1: The last three sentences of the section are “In Figure 1, a clear difference can be observed between SARG, ARGminer and MEGARes databases in contrast with NDARO, ResFinder and CARD in the relationship between the number of unique sequences and corresponding genes. The major differences between the two groups is that CARD, NDARO and ResFinder are primer databases mainly based on the extensive review of the corresponding scientific literature [37], while the others are assembled from other data resources. One might expect that with keeping one reference sequence for each gene, these databases are prone to producing false negatives in homology searches; however, this is overcome in CARD with the use of individual detection threshold for genes stored in the Model Ontology [36] and the use of HMMs (Hidden Markov Models) in the detection software of NDARO when no high confidence match can be retrieved [34].” Based on the errors that we have found, we have modified it to the following: “In Figure 1, a clear difference can be observed between CARD and the rest of the databases in the relationship between the number of unique sequences and corresponding genes. One might expect that with keeping one reference sequence for each gene, CARD is prone to producing false negatives in homology searches; however, this is overcome in CARD with the use of individual detection threshold for genes stored in the Model Ontology [36].”

4. Section 3.3: In the first sentence of the second paragraph, the number of genes with no species classification in the CARD database has been revised from 59 to 19.

5. Section 3.3: The fourth sentence of the second paragraph of this section was changed from “CARD stores 10 genes for *Chlamydomonas* algae and five for the archaea genus *Halobacterium*, while PointFinder has six genes for *Plasmodium* protozoa.”, to “CARD stores two genes for *Chlamydomonas* algae and two for the archaea genus *Halobacterium*, while PointFinder has six genes for *Plasmodium* protozoa.”

6. Section 3.3: In the seventh sentence of the second paragraph of this section, the number of AMR genes assigned to the genus *Mycobacterium* in CARD has been changed from 1168 to 63.

7. Section 3.3: The last sentence of the third paragraph of this section was changed from “An especially high number of genes is stored in the database for the *Mycolicibacterium* genus (37 genes), which is in the forefront as a potential bacterium for degrading plastic pollutants [60].” to the following: “A notable number of genes is stored in the database for the *Mycolicibacterium* genus (4 genes), which is in the forefront as a potential bacterium for degrading plastic pollutants [60].”

## Error in Figure

Due to the errors stated above, Figure 1, Figure 2 and Figure 3 and Supplementary Figure S2 has been updated.

In Figure 1, the number of sequences has been changed from 6035 to 6092 and from 3146 to 3137 for the NDARO and ResFinder databases, respectively. Furthermore, the number of resistance genes has been changed to 4898 and 2532 for the NDARO and ResFinder databases, respectively.

In Figure 2 and Supplementary Figure S2, the number of genes for each antimicrobial group has been recalculated for the NDARO and ResFinder databases. Additionally, the missing categories (Rhodamine, Disinfecting agents and intercalating dye and Acridine dye) has been added to the antimicrobial groups in the CARD database.

In Figure 3, the number of genes with resistance through mutations stored for the CARD and NDARO databases has been recalculated.

The authors apologize for any inconvenience caused and state that the scientific conclusions are unaffected. This correction was approved by the Academic Editor. The original publication has also been updated.

## Figures and Tables

**Figure 1:**
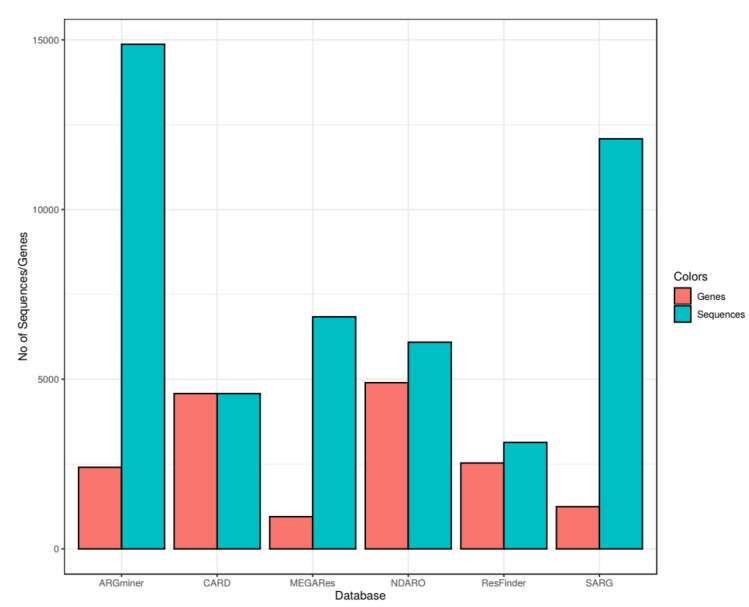
ARG and sequence content of the databases. Only antibiotic and biocide resistance genes were considered for the plot. For each database on the x axis, the number of unique sequences and the corresponding number of unique genes were determined. The y axis represents the number of genes and sequences. Red bars show the gene number while blue bars represent the number of sequences stored in each database.

**Figure 2:**
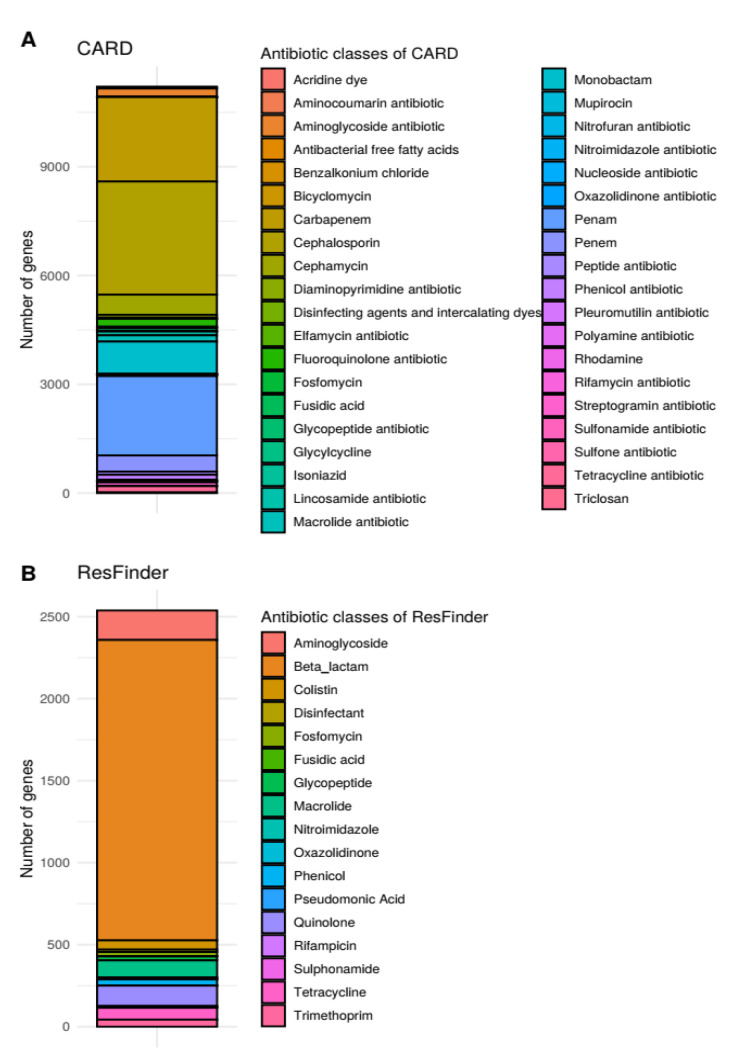
Number of unique genes for each antibiotic class stored in CARD and ResFinder. Bars represent the number of genes in each unique antibiotic or biocide categories, where colors are associated with the specific antibiotics themselves. As one gene can confer resistance to multiple antibiotics, it is possible that the same gene is counted for two or more antibiotics. The plots show the data for CARD (**A**) and ResFinder (**B**), respectively.

**Figure 3:**
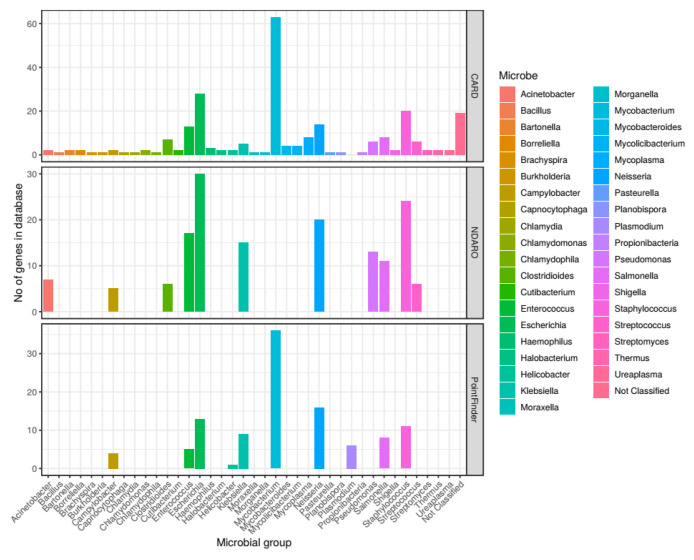
Number of genes conferring resistance through mutations for each microbial genus in CARD, NDARO and MEGARes databases. Genes conferring resistance through mutations was calculated at the genus level for each microbe stored in each database. Only one group could not be summarized at the genus level (propionibacteria). Microbial genus is on the x axis and the number of genes associated with each group in the database is represented by the y axis. Rows show the data separately for each database. Columns are colored by the microbial genus.

**Figure S2:**
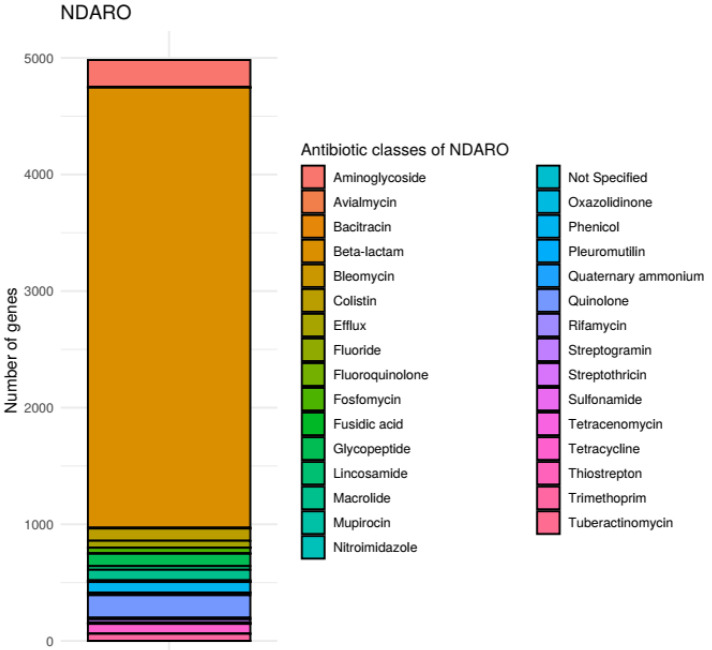
NDARO gene counts of each antibiotic class. Number of resistance genes were determined for each class of antibiotics stored in the NDARO database. Only antibiotic- and biocid resistance genes were considered for this plot.
