# Supplementary material for: Review and Comparison of Antimicrobial Resistance Gene Databases
Source: Antibiotics (Basel). 2022 Mar 4;11(3):339. doi: 10.3390/antibiotics11030339 (PMC8944830; doi:10.3390/antibiotics11030339)
Supplement: Supplementary file 1 [file antibiotics-11-00339-s001.zip › Supplementary_File_1.pdf]

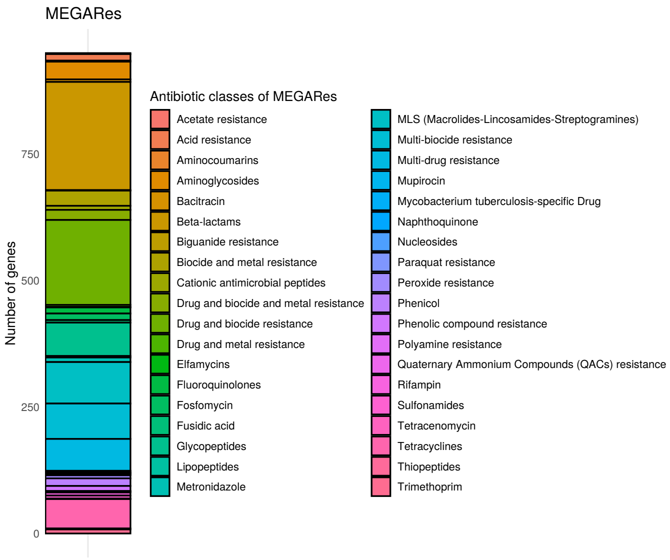

Supplementary Figure S1: MEGARes gene counts of each antibiotic class. Number of resistance genes were determined for each class of antibiotics stored in the MEGARes database. Only antibiotic- and biocid resistance genes were considered for this plot.

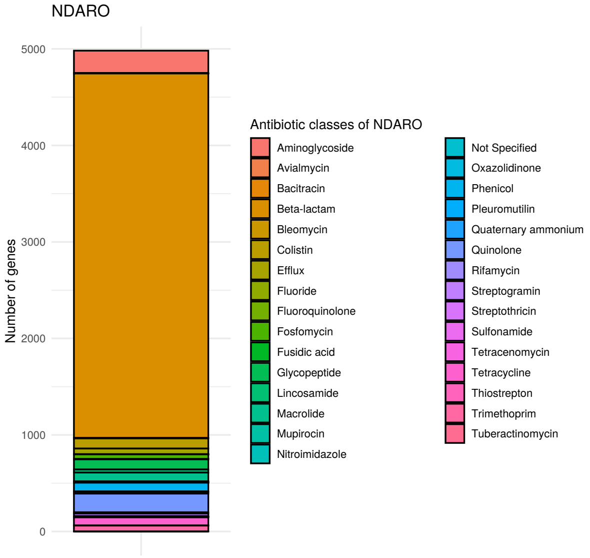

Supplementary Figure S2: NDARO gene counts of each antibiotic class. Number of resistance genes were determined for each class of antibiotics stored in the NDARO database. Only antibiotic- and biocid resistance genes were considered for this plot.

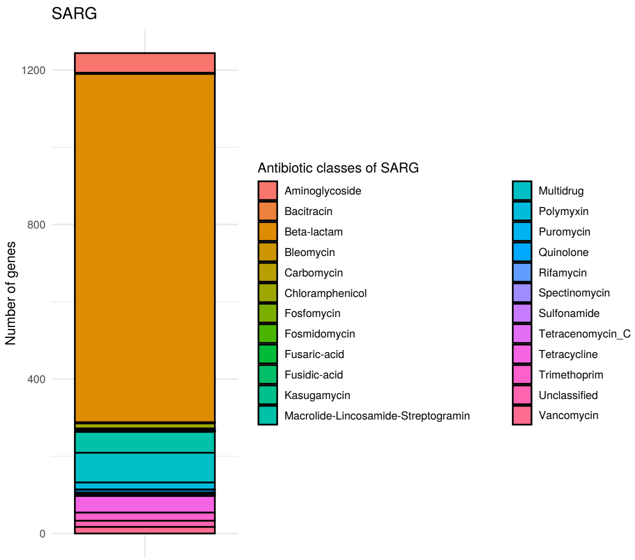

Supplementary Figure S3: SARG gene counts of each antibiotic class. Number of resistance genes were determined for each class of antibiotics stored in the SARG database. Only antibiotic- and biocid resistance genes were considered for this plot.
